# Role of mitochondrial DNA damage and dysfunction in veterans with Gulf War Illness

**DOI:** 10.1371/journal.pone.0184832

**Published:** 2017-09-14

**Authors:** Yang Chen, Joel N. Meyer, Helene Z. Hill, Gudrun Lange, Michael R. Condon, Jacquelyn C. Klein, Duncan Ndirangu, Michael J. Falvo

**Affiliations:** 1 War Related Illness and Injury Study Center, Veterans Affairs New Jersey Health Care System, East Orange, New Jersey, United States of America; 2 New Jersey Medical School, Rutgers Biomedical and Health Sciences, Newark, New Jersey, United States of America; 3 Nicholas School of the Environment, Duke University, Durham, North Carolina, United States of America; 4 Pain and Fatigue Study Center, Beth Israel Medical Center and Albert Einstein Medical Center, New York, New York, United States of America; 5 Surgical Services, Veterans Affairs New Jersey Health Care System, East Orange, New Jersey, United States of America; Technion Israel Institute of Technology, ISRAEL

## Abstract

Gulf War Illness (GWI) is a chronic multi-symptom illness not currently diagnosed by standard medical or laboratory test that affects 30% of veterans who served during the 1990–1991 Gulf War. The clinical presentation of GWI is comparable to that of patients with certain mitochondrial disorders–i.e., clinically heterogeneous multisystem symptoms. Therefore, we hypothesized that mitochondrial dysfunction may contribute to both the symptoms of GWI as well as its persistence over time. We recruited 21 cases of GWI (CDC and Kansas criteria) and 7 controls to participate in this study. Peripheral blood samples were obtained in all participants and a quantitative polymerase chain reaction (QPCR) based assay was performed to quantify mitochondrial and nuclear DNA lesion frequency and mitochondrial DNA (mtDNA) copy number (mtDNAcn) from peripheral blood mononuclear cells. Samples were also used to analyze nuclear DNA lesion frequency and enzyme activity for mitochondrial complexes I and IV. Both mtDNA lesion frequency (*p* = 0.015, *d* = 1.13) and mtDNAcn (*p* = 0.001; *d* = 1.69) were elevated in veterans with GWI relative to controls. Nuclear DNA lesion frequency was also elevated in veterans with GWI (*p* = 0.344; *d* = 1.41), but did not reach statistical significance. Complex I and IV activity (*p* > 0.05) were similar between groups and greater mtDNA lesion frequency was associated with reduced complex I (*r*^*2*^ = -0.35, *p* = 0.007) and IV (*r*^*2*^ = -0.28, *p* < 0.01) enzyme activity. In conclusion, veterans with GWI exhibit greater mtDNA damage which is consistent with mitochondrial dysfunction.

## Introduction

Approximately 25–32% of veterans who served during Operations Desert Storm and Shield (Gulf War) are afflicted with a chronic multisystem illness referred to as Gulf War Illness (GWI) [1]. The clinical presentation of GWI is heterogeneous, though characterized predominantly by fatigue, widespread pain, exercise intolerance and cognitive dysfunction. As symptoms of GWI span multiple high-energy systems [2, 3], mitochondrial dysfunction appears biologically plausible. Patients with mitochondrial disorders present with multisystem symptoms displaying clinical heterogeneity and tissue-specific manifestations [4]. Tissues and organs that rely predominantly on oxidative phosphorylation for energy production are often those that exhibit the greatest pathology when mitochondrial function is compromised [5]. Despite this overlap in multi-symptom presentation and heterogeneity among patients with mitochondrial disorders and veterans with GWI, only two recent studies have investigated mitochondrial dysfunction in GWI [6]. Specifically, Koslik et al. measured post-exercise phosphocreatine recovery in skeletal muscle–an indirect measure of mitochondrial capacity–following ankle flexion exercise in veterans with GWI and observed prolonged phosphocreatine recovery in comparison to controls [6]. In addition, Abdullah et al. reported alterations of certain plasma mitochondrial lipids (i.e., acylcarnitine) among veterans with GWI [7]. Therefore, whether mitochondrial dysfunction is involved in the pathobiology GWI remains to be established but is under active investigation.

Mitochondrial dysfunction has previously been hypothesized as an explanation for symptoms underlying GWI, particularly in the contexts of environmental exposures during deployment (e.g., carbamates and organophosphates) [8, 9]. Emerging evidence supports this rationale as mitochondria are increasingly recognized as a target for environmental toxicants [10, 11]. In fact, mitochondria appear uniquely susceptible to toxicants as a result of: 1) accumulation of toxicants due to the high lipid content of the mitochondrial membranes, slight negative charge of the mitochondrial matrix, and presence of metal cation transporters; 2) ability to activate organic compounds via mitochondrial cytochrome P450s; 3) the presence of the reactive oxygen species generating electron transport chain; 4) reduced repair mechanisms for mitochondrial DNA (mtDNA) in comparison to the nuclear genome, and 5) the potential for toxicant exposure to increase the endogenous level of production of reactive oxygen species [10, 12–18]. While the role of oxidative stress in generating mtDNA mutations is currently contentious [19], mtDNA is highly sensitive to oxidative damage [20], and mtDNA copy number may also be altered by oxidative stress. Specifically, increased intracellular reactive oxygen species may initially lead to mitochondrial biogenesis (i.e., increased mitochondrial content) as an adaptive response, but over time could damage mitochondrial DNA, proteins and membranes leading to mitochondrial dysfunction and mitochondrial DNA depletion [21, 22]. No studies to date have evaluated whether mitochondrial content and/or damage is elevated in veterans with GWI.

An increasing number of studies have described altered mtDNA content and damage in circulation in genetic and non-genetic diseases with associated mitochondrial dysfunction, suggesting that circulating mtDNA may serve as a marker of disease status [23]. These findings are of particular relevance to GWI, which currently relies on case definitions derived from self-reported symptoms [24, 25]. In light of the clinical presentation of veterans with GWI and the susceptibility of mitochondria to environmental toxicants, we hypothesize that GWI is maintained, in part, by mitochondrial dysfunction that is detectable by elevated levels of mtDNA content and damage in circulation. Therefore, the primary goal of this preliminary study is to examine markers of mitochondrial damage and function in veterans with GWI relative to controls. Specifically, we utilized a quantitative polymerase chain reaction (QPCR)-based assay for the quantification of both mitochondrial and nuclear DNA damage [26], which has previously been used to identify mtDNA damage in the blood of patients with known mitochondrial dysfunction (i.e., Friedreich’s ataxia) [23].

## Materials and methods

### Participants

Twenty-eight participants volunteered to participate in this study, including 21 cases of GWI (GWI+) and 7 controls (GWI-). Case status was assigned using the Centers for Disease Control (CDC) and Kansas criteria [27]. In brief, cases must endorse moderate-to-severe symptoms in ≥ 3 domain areas (i.e., fatigue, pain, neurological/cognitive/mood, skin, gastrointestinal and respiratory) that began after 1990 and persisted for ≥ 1 year. Comorbid conditions (i.e., diabetes, heart disease, stroke, lupus, multiple sclerosis, cancer, etc.) that may account for chronic symptoms were excluded per case definition [25]. Control participants consisted of three veterans and four civilians who did not meet the CDC or Kansas criteria and were also free from chronic infection, neurologic, endocrine, or cardiovascular disease. Additional details on screening procedures and exclusions are provided in supporting information (Table A in S1 Text). Participant characteristics are provided in Table 1. Self-reported fatigue severity and physical health-related functioning were also assessed in all participants via the Fatigue Severity Scale [28] and the Veterans version of the Short Form 36 Health Survey [29], respectively. All participants provided their informed written consent, and procedures were reviewed and approved by the Department of Veterans Affairs New Jersey Health Care System’s Institutional Review Board.

**Table 1:** Participant characteristics and self-reported symptoms for cases with (GWI+) and without (GWI-) Gulf War illness.

	Cases (GWI+) n = 21	Controls (GWI-) (n = 7)
**Age (years)**	49.8 ± 5.1	51.3 ± 4.9
**Sex (female/male)**	2/19	2/5
**Ethnicity**		
Hispanic or Latino	23.8%	14.3%
Not Hispanic or Latino	42.9%	71.4%
Unknown	33.3%	14.3%
**Race**		
American Indian or Alaska Native	4.8%	14.3%
Asian	4.8%	-
Black or African American	19.0%	28.6%
Native Hawaiian or Pacific Islander	-	-
White	71.4%	42.9%
Unknown	-	14.3%
**Body Mass Index (kg/m**^**2**^**)**	30.2 ± 4.3	30.4 ± 4.5
**Physical Activity (min·wk**^**-1**^**)**	124.5 ± 190.8	172.5 ± 217.2
**Smoking History (pack-years)**	6.3 ± 10.9	14.5 ± 14.8
**Fatigue Severity Score**†	48.3 ± 11.3	21.6 ± 7.5
**Physical Composite Score**‡	38.3 ± 10.2	58.8 ± 4.5
Kansas GWI Screening Domains*		
**Fatigue**	8.0 ± 3.3	2.0 ± 2.5
**Pain**	3.6 ± 1.8	0.17 ± 0.4
**Neurological/Cognitive/Mood**	18.0 ± 10.0	3.3 ± 6.8
**Skin**	2.2 ± 1.8	0.0 ± 0.0
**Gastrointestinal**	3.7 ± 3.1	0.0 ± 0.0
**Respiratory**	1.6 ± 1.9	0.0 ± 0.0

Data presented as mean ± standard deviation.

†Fatigue severity scores ≥ 35 are considered clinically fatigued

‡Physical composite scores ≤ 50 are reflective of poorer physical health-related functioning

*Symptom score totals were computed for each domain of Kansas GWI Questionnaire.

### Peripheral blood sample acquisition

Peripheral blood samples (15 mL) were obtained via venipuncture and collected into vacutainer tubes containing di-potassium EDTA salt. Peripheral blood mononuclear cells (PBMCs) and plasma were separated from whole blood via a commercial solution of polysucrose and sodium diatrizoate (Histopaque®-1077, Sigma Aldrich) and centrifugation at 700 *g* for 30 min. Plasma was aliquoted into microcentrifuge tubes and stored at -80°C. PBMCs were twice washed with phosphate-buffered saline (1xPBS, pH 7.4) and centrifuged at 200 *g* for 10 min, and suspended in 100–200 µl of either 1xPBS or radioimmunoprecipitation assay (RIPA) buffer without detergent, but with protease and phosphatase inhibitor and stored at -80°C until further analysis.

### DNA isolation and quantification

PBMCs in RIPA buffer were used to extract total DNA using a commercial DNA purification kit according to the manufacturer’s protocol (QIAGEN Genomic Tip 20/G). DNA samples were quantified in a 96-well plate reader (Synergy NEO HTS; BioTek Instruments) via a standardized protocol using a fluorescence dye (Quant-iT^TM^ PicoGreen® dsDNA reagent; Molecular Probes) [30]. In brief, lambda (λ)/*Hin*dIII DNA (Invitrogen) was diluted in 1xTE buffer to a series of concentrations (0, 2.5, 5, 10, 20 ng/µL) to generate a standard curve for DNA quantification. Concentrated DNA samples were quantified and diluted to a final concentration of 3 ng/μL via serial dilution (10 ng/µL, 5 ng/µL, 3 ng/µL) and stored at -80°C for the QPCR assay.

### QPCR-based DNA damage assay

Mitochondrial DNA damage was measured using a QPCR-based assay [31] based on the principle that DNA damage will attenuate or halt DNA polymerase progression resulting in a smaller PCR product for samples with greater damage. This standardized protocol [31] was slightly modified to use KAPA^TM^ LongRange HotStart PCR kit (KapaBioSystems), detailed procedures and validation experiments are provided in supporting information (S1 Text). QPCR products were quantified using Picogreen dye in a 96-well plate reader. DNA lesion frequency (i.e., damage) was calculated for both mtDNA and nuclear DNA following a Poisson equation [f(x) = e^-λ^ λ^x^ /x!, where λ is the average lesion frequency for the non-damaged template (i.e., the zero class; x = 0, f(0) = e^-λ^)], as previously described [26]. The amplification of case (GWI+) samples (A_GWI+_) was compared to the amplification of non-damaged controls (A_GWI-_; zero class) resulting in a relative amplification ratio. The DNA lesion frequency was determined as λ = -ln (A_GWI+_/A_GWI-_). The amplification of the short mtDNA fragment is assumed to be free of lesions due to its short size and represents mtDNA copy number (mtDNAcn). The lesion frequency in mtDNA was calculated by normalizing amplification of the long mtDNA fragment to the short mtDNA fragment.

### Mitochondrial complex I and IV activities

PBMCs were also used to measure mitochondrial complex I and IV enzyme activities in a 96-well format using commercial kits according to manufacturer’s protocol (ABCAM). In brief, protein concentration of each sample was determined by standard BCA protein assay (Pierce BCA Protein Assay Kit; Thermo Scientific), and adjusted to 2.8 µg/µL and 2.5 µg/µL with 1x PBS for complex I and IV enzyme activity assays, respectively, depending on the size of available sample. Complex I activity was determined by following the oxidation of NADH (nicotinamide adenine dinucleotide) to NAD^+^ and the simultaneous reduction of a dye which lead to increased absorbance at 450 nm. Complex IV activity was determined by following the oxidation of reduced cytochrome c to decreased absorbance at 550 nm. Analysis was performed within the 96-well plate reader using kinetics protocol for complex I and IV (Gen5 Data Analysis Software). Complex I and IV activities were normalized to mtDNAcn in order to express activity as a function of mitochondrial content (S1 Text).

### Statistical analysis

Between-group differences (GWI+ vs. GWI-) for our primary outcome variables (i.e., mtDNA and nuclear DNA lesion frequency, and mtDNAcn) were assessed using the Welch’s t-test to account for unequal variances [32] with statistical significance set at p < 0.05 (two-sided). Cohen’s *d* was used to assess the magnitude of between-group differences. Post-hoc exploratory analyses were performed to compare between-group differences in enzyme activities as well as determine their association (Pearson’s correlation coefficient) with indices of mitochondrial content and damage. All statistical analyses were performed using SPSS (v.24).

## Results

**Fig 1:**
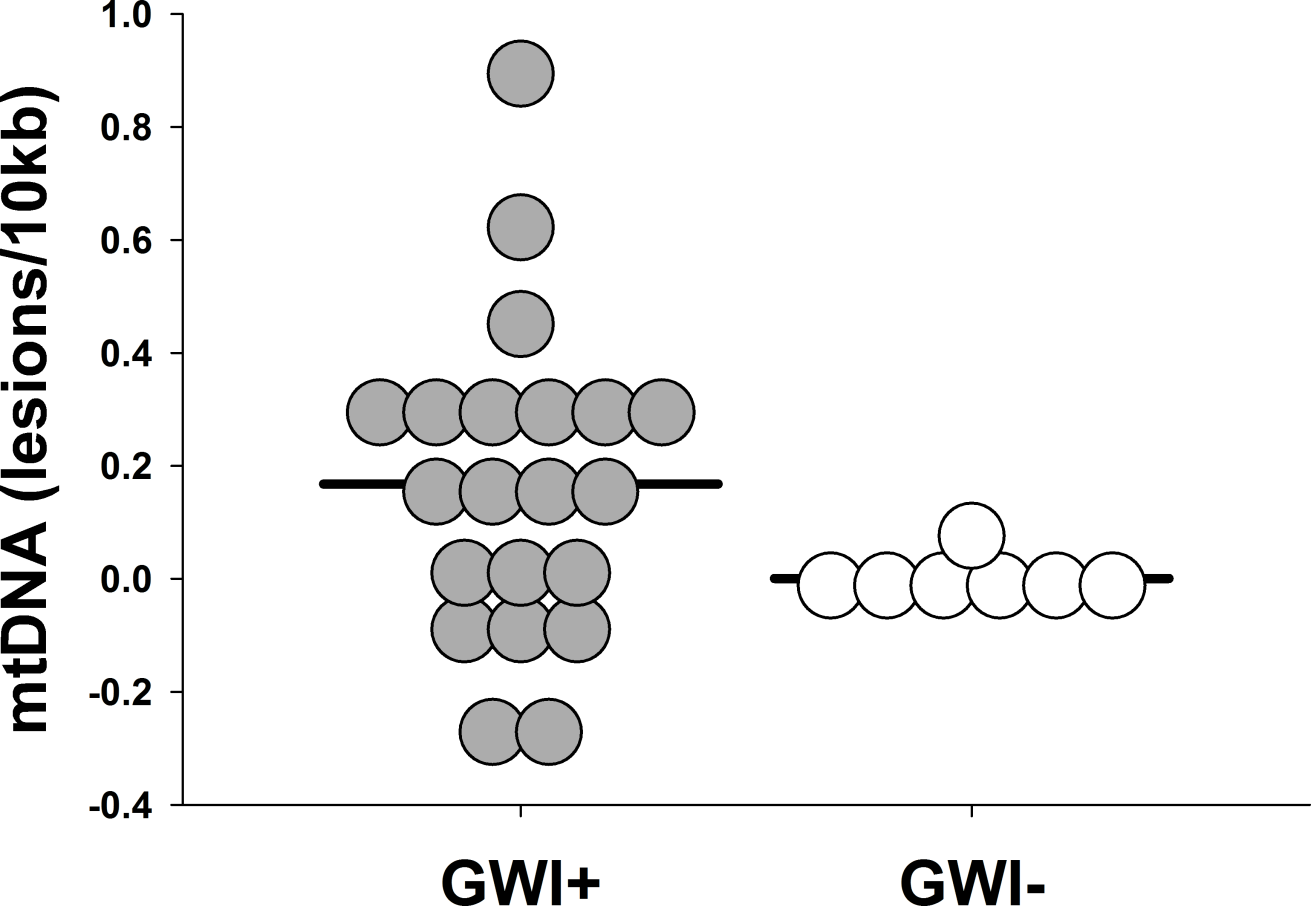
Mitochondrial DNA damage identified by QPCR analysis of blood for cases with (n = 21, GWI+) and without (n = 7, GWI-) Gulf War Illness. Dot density plot data represent the number of excess lesions found per 10 kb of DNA from mtDNA genomes in GWI+ cases as compared to controls (GWI-). Greater lesions (0.17 lesions/10 kb) were observed among cases with GWI+ relative to controls (*p* = 0.015, *d* = 1.13).

**Fig 2:**
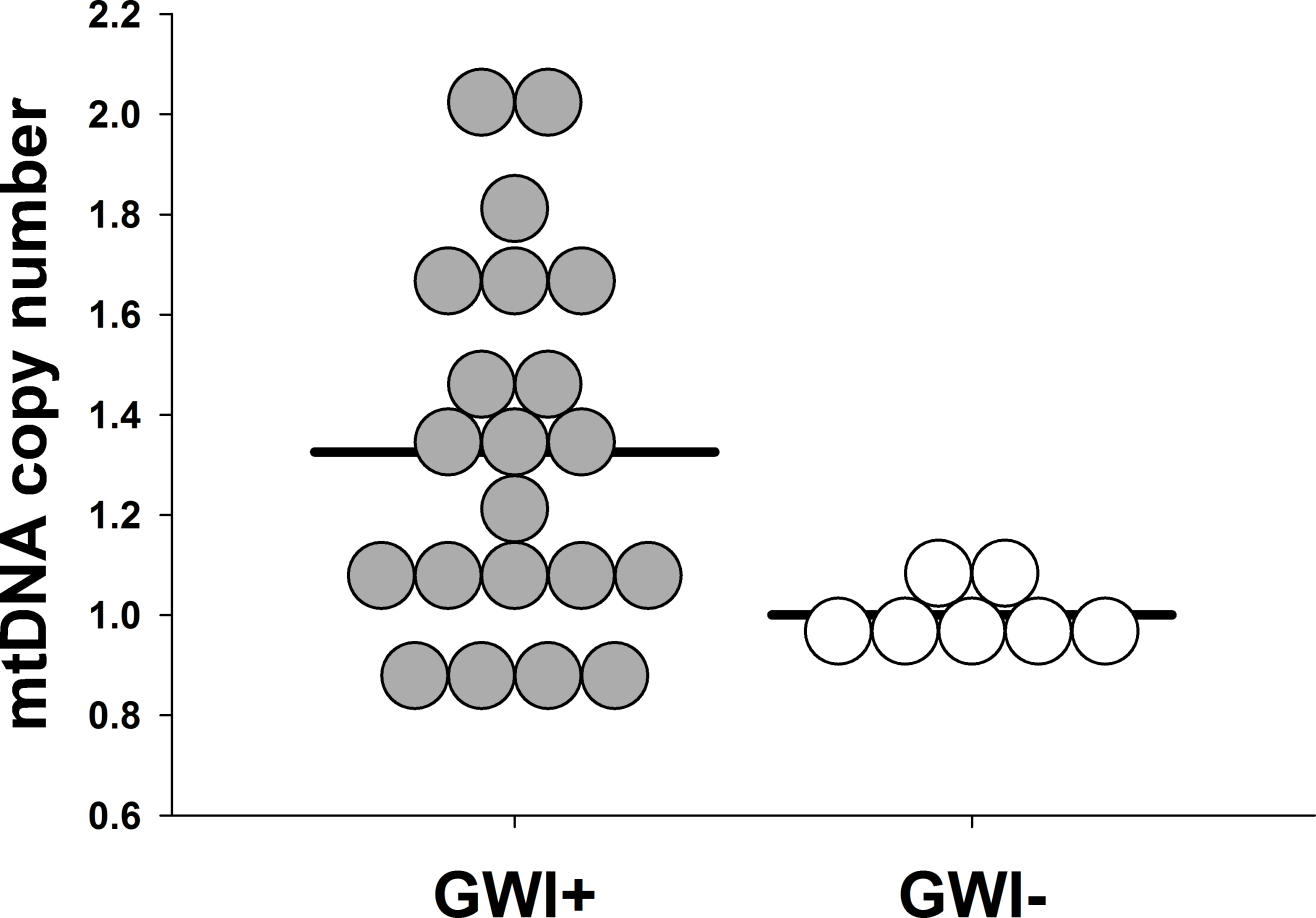
Mitochondrial content identified by QPCR analysis of blood for cases with (n = 21, GWI+) and without (n = 7, GWI-) Gulf War illness. Dot density plot data represent mitochondrial content, as indexed by mtDNA copy number, in GWI+ cases and controls (GWI-). Greater mtDNA copy number was observed among cases with GWI+ relative to controls (*p* = 0.001, *d* = 1.69).

**Fig 3:**
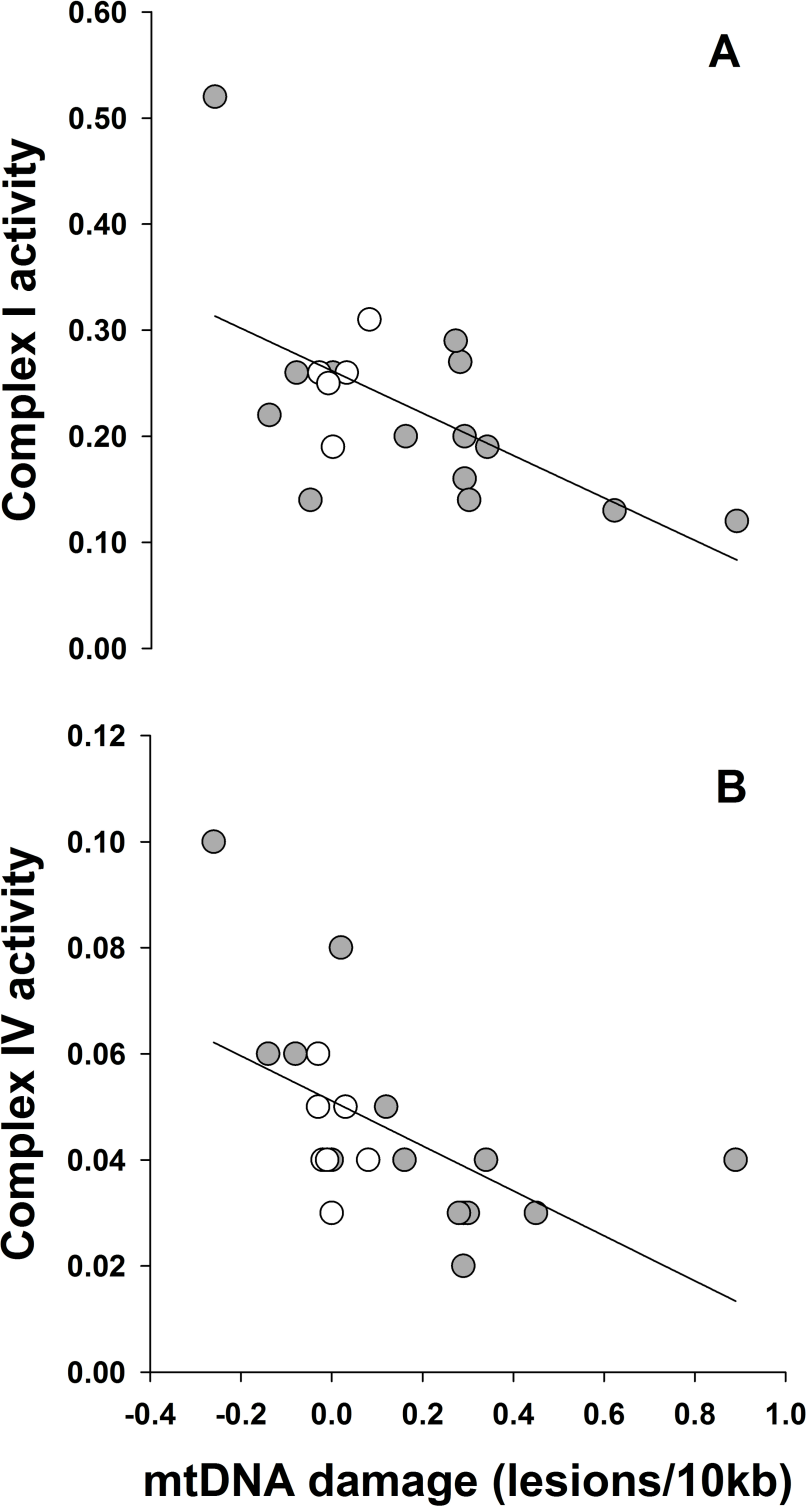
Mitochondrial DNA damage is associated with reduced Complex I and IV enzyme activity. Mitochondrial complex I activity (A) and complex IV activity (B) for cases with GWI+ (filled circles) and controls (open circles) were plotted against mtDNA lesion frequency yielding correlation coefficients of r^2^ = 0.35 and 0.30 (*p* < 0.01), respectively.

We recruited 21 veterans with GWI+ and 7 controls, whose characteristics and self-reported symptoms are presented in Table 1. In comparison to controls, veterans with GWI+ had greater mtDNA lesions (GWI+ vs. GWI-, mean ± SD: 0.17 ± 0.28 vs. 5.6 x 10^−4^ ± 0.04 lesions/10kb, *p* = 0.015, *d* = 1.13; Fig 1), mtDNAcn (1.33 ± 0.37 vs. 1.00 ± 0.05, *p* = 0.001, *d* = 1.69; Fig 2), and nuclear DNA lesions (0.06 ± 0.17 vs. 0.01 ± 0.11 lesions/10kb, *p* = 0.344, *d* = 1.41). Protein concentrations were insufficient to analyze enzyme activities for all participants; therefore, 9 individuals were excluded for complex I (GWI+ = 14, GWI- = 5) and 7 individuals were excluded for complex IV (GWI+ = 14, GWI- = 7). Enzyme activity for complex I (0.22 ± 0.10 vs. 0.25 ± 0.04, *p* = 0.357, *d* = 1.18) was reduced in GWI+, and complex IV activity (0.05 ± 0.02 vs. 0.04 ± 0.01, *p* = 0.583, *d* = 0.34) was similar between groups. Greater mtDNA lesions were associated with lower complex I (Fig 3A, n = 19, *r*^*2*^ = 0.35, *p* = 0.007) and IV activity (Fig 3B, n = 21, *r*^*2*^ = 0.30, *p* = 0.009). Similarly, greater mtDNAcn was associated with complex IV activity (n = 21, *r*^*2*^ = 0.28, *p* < 0.01), but not with complex I (n = 19, *r*^*2*^ = 0.19, *p* = 0.064).

## Discussion

This study provides the first direct biological evidence of mtDNA damage in the blood of veterans with GWI. Greater mtDNA damage and mtDNAcn are consistent with mitochondrial dysfunction [21], which may contribute to symptoms of GWI as well as the persistence of this illness over time. Levels of nuclear DNA lesion frequency were also elevated in GWI, despite prior work demonstrating that damage is more severe and persists longer in mtDNA than nuclear DNA [20]. Given increases in both mtDNA lesion frequency and mtDNAcn, we interpret these findings as evidence that mitochondrial dysfunction is involved in the pathobiology of GWI.

Circulating markers of mtDNA damage and mtDNAcn have previously been utilized for evaluating mitochondrial dysfunction secondary to environmental and chemical toxicants [33–37], which in some cases preferentially target the mitochondria [10, 11]. Baccarelli and colleagues have identified that pro-oxidant environmental exposures, i.e., ambient particulate matter, damage the mitochondrial genome which, in turn, may intensify oxidative stress [38, 39]. These findings are relevant for GWI as ambient particulate matter levels in the Persian Gulf region are up to 10-fold greater than urban cities in the United States [40], although the composition of the PM may not be the same. Despite high levels of ambient particulate matter, exposure to carbamates and organophosphates (e.g., pyridostigmine bromide, pesticides, and/or insect repellant) deserve greater attention in the context of GWI [41–43] as these agents were widely administered during the Gulf War [44, 45]. Further, self-reported exposure to some types of exposures, such as pyridostigmine bromide and pesticides, has demonstrated an association with symptom severity in a dose-response manner [46]. Similarly in our sample, self-reported exposure to pyridostigmine bromide and pesticides during deployment were reported in 76.2% and 66.7% of our GWI+ cases, respectively. In related toxicology research, studies have observed increased mtDNA damage, mtDNAcn and/or increased oxidative stress following exposure to pesticide, pyridostigmine bromide, or their combination [47–52]. Though we observed increased mtDNA damage (Fig 1) and mtDNAcn (Fig 2) in circulation of veterans with GWI, the present study was not designed to verify past exposures or confirm causality.

Mitochondrial dysfunction among veterans with GWI may help explain, in part, the persistence of this illness for over 25 years. For example, chemical and environmental exposures during deployment may have provided the initial insult to mtDNA and accumulation of damage. Damaged mtDNA may subsequently impact the efficiency of electron transport chain complexes and activity, resulting in enhanced reactive oxygen species and further damage of mtDNA [20]. In the present study, mtDNA lesions were weakly associated with enzyme activity of complexes I and IV (Fig 3), which may suggest greater mtDNA damage is associated with a reduction in mitochondrial function. This is an expected outcome of mtDNA damage, in particular in the context of the vicious cycle theory of mtDNA damage and oxidative stress, but to our knowledge is not one that has been tested in people [20]. Exploring GWI through the lens of mitochondrial genetics may also provide an understanding of why chronic symptoms among deployed Gulf War veterans are not a universal finding. For example, certain mitochondrial haplogroups are known to offer protection for specific neurodegenerative diseases [53]. In addition, Wittkopp et al. [54] recently demonstrated that individuals with mitochondrial haplogroup U may be conferred protection from traffic-related air pollution exposure. Future studies are necessary to determine whether mitochondrial genetic background confers protection from GWI, but appear warranted.

Our findings support recent work that demonstrated impaired skeletal muscle mitochondrial capacity (i.e., prolonged phosphocreatine [PCr] recovery) in veterans with GWI as detected by ^31^P magnetic resonance spectroscopy [6]. PCr recovery time is suggested to provide a robust measure of skeletal muscle oxidative capacity [55, 56], but is largely impacted by the availability and supply of oxygen [57]. Therefore, delayed PCr recovery may not solely reflect mitochondrial dysfunction. More recently, Abdullah and colleagues reported alterations of mitochondria-specific lipids (i.e., acylcarnitines) in plasma of veterans with GWI [7]. Increased plasma acylcarnitine levels are observed in response to incomplete fatty acid oxidation [58], and abnormal acylcarnitine profiles have been reported in patients with mutations in nuclear-encoded or mtDNA [59]. Elevations of plasma acylcarnitines in veterans with GWI [7] are therefore intriguing in the context of the present study’s findings of damaged mtDNA, particularly as increased plasma acylcarnitine levels have been shown to cause oxidative stress [60]. Lastly, in a randomized, placebo-controlled trial of coenzyme Q10 supplementation, Golomb et al. observed greater self-reported physical function and health in veterans with GWI who received supplementation [61].

It warrants attention that self-reported smoking history was moderately higher in our control group relative to cases (Table 1; *p* = 0.13, *d* = 0.63). This is important as individuals with greater smoking histories (i.e., > 10 pack-years) have 5-fold greater mtDNA damage than those with modest smoking histories (i.e., < 5 pack-years) [62]. Despite this, veterans with GWI+ still demonstrated an excess mtDNA lesion frequency (0.17 lesions/10kb; Fig 1) relative to controls. As lesion frequency is calculated as a relative amplification ratio [31], our observed levels of mtDNA damage in veterans with GWI+ may in fact be underestimated.

A complementary goal of the present study was to identify a circulating marker of mitochondrial dysfunction that could distinguish those with and without GWI. Though focus on peripheral blood may be considered a limitation, examining mitochondria in distinct tissues (i.e., skeletal muscle) may not be ideal in a clinically heterogeneous disorder affecting multiple organ systems. For example, current case definition criteria may identify a veteran as having GWI even in the absence of musculoskeletal symptoms; therefore, analyses restricted to one organ system may not fully capture pathology in a multisystem illness. Further, given increasing attention to mitochondrial translational research and rapid clinical tests to detect abnormal bioenergetics in mononuclear cells [63], a blood based test may be more widely implemented in the clinic. We are currently performing additional studies examining functional implications of mtDNA damage in veterans with GWI using blood-based markers for these reasons. In addition, future studies should include larger sample sizes as well additional experiments to characterize downstream effects of mitochondrial dysfunction including DNA repair capacity.

In summary, the present study evaluated the integrity of mtDNA by using a QPCR-based assay that afforded a direct and objective tool for assessing mtDNA damage. From these detailed experiments, we found that mtDNA damage is 20% greater in veterans with GWI than controls, and this mtDNA damage was associated with reduced enzyme activities in both complexes I and IV. Future studies are necessary to confirm our findings; however, this work suggests that mtDNA damage may serve as an objective biomarker of GWI. Results from the present study, along with recent work from other laboratories [6, 7], suggest mitochondrial dysfunction is involved in the pathobiology of GWI and should continue to be actively investigated.

## Supporting information

S1 Text **Figure A. Dose-response relationship between ultraviolet C radiation and mtDNA lesions. Table A. Participant exclusions.** Fifty-five eligible participants were screened for inclusion into the present study, and approximately 49% were disqualified for reasons described in the table. **Table B. PCR Conditions.** All the primer sets were produced by Integrated DNA Technology. ^a^Using primers 5999 and 14841, this reaction generated a 8.9-kb fragment from mtDNA, ^b^Using primers 48510 and 62007, this reaction generated a 13.5-kb fragment from beta-globin.
(DOCX)
