# Correction: Role of mitochondrial DNA damage and dysfunction in veterans with Gulf War Illness

**DOI:** 10.1371/journal.pone.0186711

**Published:** 2017-10-16

**Authors:** Yang Chen, Joel N. Meyer, Helene Z. Hill, Gudrun Lange, Michael R. Condon, Jacquelyn C. Klein, Duncan Ndirangu, Michael J. Falvo

The following information is missing from the Funding section: This work was supported by the Department of Veterans Affairs Clinical Science Research and Development Service (1I21CX000797). The contents do not represent the views of the U.S. Department of Veterans Affairs or the United States Government.
