# Supplementary material for: Role of mitochondrial DNA damage and dysfunction in veterans with Gulf War Illness
Source: PLoS One. 2017 Sep 14;12(9):e0184832. doi: 10.1371/journal.pone.0184832 (PMC5599026; doi:10.1371/journal.pone.0184832)
Supplement: S1 Text — Figure A. Dose-response relationship between ultraviolet C radiation and mtDNA lesions. Table A. Participant exclusions. Fifty-five eligible participants were screened for inclusion into the present study, and approximately 49% were disqualified for reasons described in the table. Table B. PCR Conditions. All the primer sets were produced by Integrated DNA Technology. aUsing primers 5999 and 14841, this reaction generated a 8.9-kb fragment from mtDNA, bUsing primers 48510 and 62007, this reaction generated a 13.5-kb fragment from beta-globin. (DOCX) [file pone.0184832.s001.docx]

**Supporting Information**

**Participant Screening**

In order to ensure participants met the inclusion and exclusion criteria for this study, we conducted pre-study screening over the phone and in-person, and supplemented this screening with electronic medical record review when available. Of the 55 participants screened for this study, 28 were included in this study. The reason for exclusion is provided in Table A.

| **Reason for Exclusion** | **Total Number** |
| --- | --- |
| Heart disease | 6 |
| Cancer | 4 |
| Hepatitis | 3 |
| Diabetes | 3 |
| Multiple Sclerosis | 2 |
| Neurological Disorder/Disease | 2 |
| Morbid obesity | 2 |
| Pulmonary disease | 1 |
| Rheumatologic disease | 1 |
| Psychiatric disease | 1 |
| Stroke | 1 |
| High-dose mitochondrial therapy | 1 |
|  |  |
| Total excluded | 27 |

**Table A. Participant exclusions.** Fifty-five eligible participants were screened for inclusion into the present study, and approximately 49% were disqualified for reasons described in the table.

**QPCR-based DNA Damage Assay**

The present study evaluated mitochondrial DNA (mtDNA) and nuclear DNA lesion frequency in human peripheral blood mononuclear cells (PBMCs) using a standard QPCR-based assay [1] via KAPA^TM^ LongRange HotStart PCR kit (KapaBioSystems, *cat. no. KK3502*). In order to confirm the accuracy and sensitivity of this QPCR technique in our study, we utilized ultraviolet C radiation as a model genotoxin [2] to generate DNA damage in our samples. The extracted DNA from mononuclear cells at a concentration of 3 ng/μL in 50 μL of liquid was treated with a serial exposure to ultraviolet C radiation (0, 5, 10, 20 J/m^2^ using an ultraviolet cross-linker), and treated DNA was used for QPCR technique described above to evaluate DNA lesion frequency in mtDNA and nuclear DNA. We observed a dose-response relationship between DNA lesion frequency (mtDNA and nuclear DNA) and irradiance of ultraviolet C radiation, which served as proof of concept for the QPCR technique in the present study (Figure A).


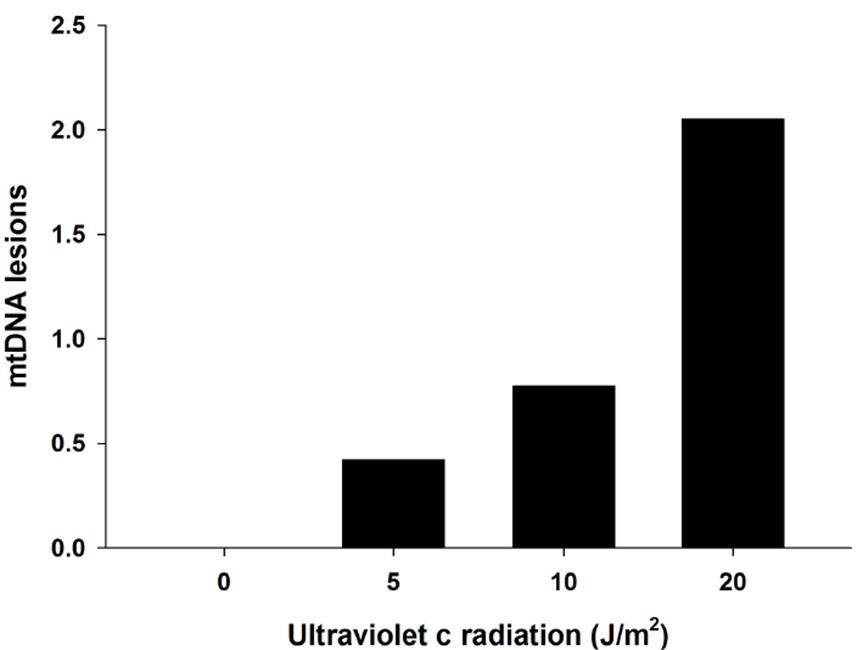


**Figure A. Dose-response relationship between ultraviolet C radiation and mtDNA lesions.**

QPCR reaction reagents were prepared in a PCR workstation (Airclean® Systems). 15 ng of DNA template (5 µL of 3 ng/µL DNA sample) was used for each reaction. The “50% control” that contained control DNA template was diluted 1:1 with 1xTE buffer, and the “blank” that contained only 1xTE buffer were used for evaluating the accuracy of each QCR reaction. Using KAPA^TM^ LongRange HotStart PCR kit, a master mix was made in following order:

1) 24.5 µL nuclease-free H_2_O (for a final volume of 50µL),

2) 10 µL of 5x buffer solution,

3) 3.5 µL of MgCl_2_ (25 mM stock, 1.75 mM final),

4) 2.5 µL of forward primer (10 mM stock, 0.5 µM final),

5) 2.5 µL of reverse primer (10 µM stock, 0.5 µM final),

6) 1.5 µL of dNTPs (10 mM each dNTP, 0.3 mM final), and

7) 0.5 µL KAPA LongRange HotStart DNA Polymerase (2.5 U/µL).

45 µL of master mix was added to each DNA template, mixed well and spun down. For each QPCR product (short mtDNA fragment [Short mito], long mtDNA fragment [Long mito], long nuclear DNA fragment [Long nuclear]), the primer sets, cycle number of amplification, annealing temperature (Tm) and time of Tm are listed in Table B.

PCR cycling conditions as follows:

1. 95^o^C for 3 minutes
2. Cycle number
   1. 95^o^C for 15 sec
   2. Tm, time of Tm
   3. *72 ^o^C for 45 sec (this step only for Short mito)*
3. 72^o^C for 10 minutes
4. 8^o^C hold

|  | **Primer** | **Sequence** | **Direction** | **Cycle** | **Tm** | **Time of Tm** |
| --- | --- | --- | --- | --- | --- | --- |
| Long Mito^a^ | 5999 | 5’-TCT AAG CCT CCT TAT TCG AGC CGA-3’ | Forward | 24 | 64 °C | 12 min |
|  | 14841 | 5’-TTT CAT CAT GCG GAG ATG TTG GAT GG-3’ | Reverse | 24 | 64 °C | 12 min |
| Short Mito | 48510 | 5’-CCC CAC AAA CCC CAT TAC TAA ACC CA-3’ | Forward | 22 | 60 °C | 45 s |
|  | 14841 | 5’-TTT CAT CAT GCG GAG ATG TTG GAT GG-3’ | Reverse | 22 | 60 °C | 45 s |
| Long Nuclear^b^ | 48510 | 5’-CGA GTA AGA GAC CAT TGT GGC AG-3’ | Forward | 28 | 62 °C | 12 min |
|  | 62007 | 5’-GCA CTG GCT TAG GAG TTG GAC-3’ | Reverse | 28 | 62 °C | 12 min |

**Table B. PCR Conditions**. All the primer sets were produced by Integrated DNA Technology. ^a^Using primers 5999 and 14841, this reaction generated a 8.9-kb fragment from mtDNA, ^b^Using primers 48510 and 62007, this reaction generated a 13.5-kb fragment from beta-globin

**Mitochondrial content**

From the QPCR-based assay described above, mtDNA copy number (mtDNAcn) was quantified and serves as an index of mitochondrial content. Using mtDNAcn as an estimate of content is recommended, particularly when the quantity of the sample is limited [3, 4]. As an internal control, we compared mtDNAcn with citrate synthase activity in remaining samples of 10 participants. Specifically, our citrate synthase assay was modified from a spectrophotometer protocol [5] and modified into a 96-well format. For each sample, 20 μg of protein from PBMCs were adjusted with deionized water to volume of 64 μL, and added to a well of a 96-well plate, which included a “blank” comprised of only 64 μL of deionized water. To analyze multiple samples, a master mix per well was prepared using the following reagents:

1. 100 μL of Tris (200 mM, pH 8.0) with Triton X-100 (0.2% (vol/vol)),
2. 20 μL of DTNB (5,5′-Dithiobis(2-nitrobenzoic acid, 100 µM, freshly made in 100 mM Tris (pH 8.0)), and
3. 6 μL of Acetyl CoA (Acetyl CoA lithium salt in deionized water, 10 mM).

Approximately 126 µL of master mix was added to each well and mixed well by shaking for 5s. The kinetics protocol was set up in our reader’s software (Gen5 Data Analysis Software ) and synced for measuring absorbance at 412 nm for 10 min, with measurement interval of 42 s. After adding 10 uL of 10 mM oxaloacetic acid (freshly made) to each well, the plate was immediately loaded into the 96-well plate reader to record the kinetics reading. In principle, this assay was based on a rate-limiting reaction catalyzed by citrate synthase (Acetyl-CoA + oxalacetate + H_2_O 🡪citrate + CoA-SH), which was coupled to an irreversible chemical reaction (CoA-SH + DTNB 🡪 TNB + CoA-S-S-TN) that leads to increased absorbance at 412 nm. Citrate synthase activity was compared to mtDNAcn for these 10 participants, and we observed a strong positive association (n = 10, *r = 0.697, p < 0.05*).

**References**

1. Gonzalez-Hunt CP, Rooney JP, Ryde IT, Anbalagan C, Joglekar R, Meyer JN. PCR-Based Analysis of Mitochondrial DNA Copy Number, Mitochondrial DNA Damage, and Nuclear DNA Damage. Current protocols in toxicology. 2016;67:20.11.1-20.11.25. Epub 2016/02/02. doi: 10.1002/0471140856.tx2011s67. PubMed PMID: 26828332; PubMed Central PMCID: PMCPMC4928199.

2. Meyer JN. QPCR: a tool for analysis of mitochondrial and nuclear DNA damage in ecotoxicology. Ecotoxicology. 2010;19(4):804-11. Epub 2010/01/06. doi: 10.1007/s10646-009-0457-4. PubMed PMID: 20049526; PubMed Central PMCID: PMC2844971.

3. Malik AN, Czajka A. Is mitochondrial DNA content a potential biomarker of mitochondrial dysfunction? Mitochondrion. 2013;13(5):481-92. Epub 2012/10/23. doi: 10.1016/j.mito.2012.10.011. PubMed PMID: 23085537.

4. Wang H, Hiatt WR, Barstow TJ, Brass EP. Relationships between muscle mitochondrial DNA content, mitochondrial enzyme activity and oxidative capacity in man: alterations with disease. Eur J Appl Physiol Occup Physiol. 1999;80(1):22-7. doi: 10.1007/s004210050553. PubMed PMID: 10367719.

5. Spinazzi M, Casarin A, Pertegato V, Salviati L, Angelini C. Assessment of mitochondrial respiratory chain enzymatic activities on tissues and cultured cells. Nat Protoc. 2012;7(6):1235-46. doi: 10.1038/nprot.2012.058. PubMed PMID: 22653162.
